# Different spatio-temporal strategies for controlling a striking gesture to slide an object toward a target distance

**DOI:** 10.1007/s00221-026-07305-x

**Published:** 2026-05-22

**Authors:** Sylvain Famié, Michel-Ange Amorim

**Affiliations:** 1 CIAMS, Inria, Université Paris-Saclay, Gif-sur-Yvette, 91190 France; 2 Laboratory of Neuromotor Physiology, Santa Lucia Foundation, Rome, 00142 Italy

**Keywords:** Spatio-temporal strategies, Sliding an object, Striking gesture, Interindividual variability, Motor Coordination

## Abstract

Humans never perform the same movement twice, and there is interindividual variability in the spatio-temporal regulation of movements. The objective of the present study was to investigate the relationship between spatio-temporal strategies and motor coordination strategies involved in controlling the impact speed during a striking gesture. The literature suggests three distinct strategies for spatio-temporal control of the ballistic strike to cope with task demand: varying strike amplitude while keeping duration constant; keeping amplitude constant while modulating duration; or adjusting both to change impact acceleration. In this study, participants (*n* = 33) struck a cube sliding toward a target distance under varying task demands. K-Means clustering and correlational analyses identified three distinct strategies, none of which matched the “constant amplitude” strategy. Regression analyses confirmed that strike duration was modulated differently by cluster as a function of impact speed. While clusters exploited strike amplitude and duration differently, impact speed was the common control variable across clusters, which did not differ in mean performance (spatial error). Hierarchical Multiple Factor Analysis validated these strategies (“Inverse Duration-Amplitude”, “Temporal Invariance”, and “Late-Impulse”) by distinguishing between-cluster differences from within-cluster variability. Dimensions 1 and 2 differentiated strategies via mean amplitude and temporal scaling rules. Conversely, Dimensions 3 and 4 captured within-cluster variability in the combined magnitude of amplitude and duration, and impact speed intensity, respectively. Results show that, despite individual differences in movement scale, participants adopt robust strategies to regulate impact speed in response to task demands. Finally, we found that spatio-temporal strategies and motor coordination are independent.

## Introduction

When we perform a striking task to slide an object toward a given distance, such as sliding a box of items during a move, or in sports like curling, we need to parameterize the strike movement to provide the object with an optimal initial speed (Famié et al. 2022). Schmidt (1975) noted that movement parameterization involves adjustments in physical parameters, such as movement time and movement amplitude, as well as the selection of the effector limb. The study by Delay et al. (1997) on golf putting tested whether players modulated force at strike impact by controlling either the amplitude of the movement while keeping its duration constant, or the duration of the movement while keeping its amplitude constant. Their results showed that participants kept the downswing duration constant and increased its amplitude to increase the club speed at ball contact (impact speed) as the distance from the target increased. This modulation of movement amplitude as a function of increasing distance was also observed in other studies of golf putting (Craig et al. 2000), whether among beginners or experts (Hume et al. 2005). Sim and Kim (2010) showed that experts and novices do not differ in the strike duration of the downswing movement as a function of target distance. However, experts demonstrate greater accuracy with lower impact velocity and smaller amplitude, reflecting better velocity transmission. In general, when the temporal duration of a movement is fixed or rhythmic, we naturally modify the amplitude of the movement to increase the force of impact (Hove and Keller 2010).

When the movement amplitude is limited, the duration of movement is regulated to perform the task (Newell et al. 1980; Schmidt et al. 1988). In sports tasks that require precise timing to strike an object, athletes often increase impact speed by reducing movement time, enabling greater speed at impact and accuracy in their performance. For example, in golf and baseball, athletes optimize the timing of their swing to accelerate the club or bat at impact, achieving maximum velocity in a brief window (Hume et al. 2005; Katsumata 2007). The reduction in movement time requires refined sensorimotor coordination and perceptual feedback, which players use to adapt timing just before impact. In baseball, participants can adjust the duration of the strike from one trial to the next based on the pitch, demonstrating the importance of visual information picked up from the pitcher’s gesture in optimizing speed at impact (Gray 2002).

Another strategy for controlling a striking gesture is to focus on acceleration before impact. Along those lines, in their studies on golf putting, Dias et al. (2021) identified a subgroup of participants who, as target distances increased, increased swing amplitude while reducing downswing duration, thereby modulating the movement’s acceleration. Dias et al. (2014) also investigated the influence of distance and slope on golf putting and showed that participants adapt their movements by adjusting task parameters, such as the duration of the arm in the backswing phase, the velocity of the golf head, and the acceleration at impact. Downswing duration and acceleration are increased in order to cope with the presence of a slope. Furthermore, Hasegawa et al. (2017) showed that one of the markers of performance for professionals compared to amateurs in golf putting is their ability to minimize acceleration variability, indicating greater precision in adjusting force.

In the present study, we aimed to identify different spatio-temporal strategies used to control the impact velocity during a striking gesture, based on three key parameters: *Strike Duration* (*D*), *Strike Amplitude* (*A*), and *Impact Speed* (*IS*). Specifically, we hypothesized that the index fingertip speed at impact can be regulated through distinct spatio-temporal strategies:

- by varying the *Strike Duration* while keeping *Strike Amplitude* constant (a negative correlation between *D* and *IS* is expected);
- by varying the *Strike Amplitude* while keeping the *Strike Duration* constant (no correlation between *D* and *IS* is expected);
- by jointly varying *Strike Amplitude* and *Strike Duration*, thereby regulating the gesture acceleration (a positive correlation between *D* and *IS* is expected).

This study extends our previous work (Famié et al. 2022), which investigated how upper-limb coordination contributes to the control of kinetic energy at impact (Biryukova et al. 2015; Bril et al. 2012) to transmit the kinetic energy required to slide toward a target distance to an object. In that study, two motor coordination strategies were identified: one group of participants increased *Impact Speed* mainly by varying forearm amplitude, whereas the other group increased *Impact Speed* mainly by varying wrist amplitude. However, the spatio-temporal control mechanisms underlying these coordination strategies were not examined. The main objective of the present study was, therefore, to investigate the relationship between spatio-temporal strategies and motor coordination strategies involved in controlling *Impact Speed* during a striking gesture. We hypothesized that participants who regulate their movement mainly through amplitude modulation would rely more on forearm rotation, whereas those who regulate it primarily through gesture duration would rely more on wrist rotation, which involves smaller amplitude variations. Furthermore, participants who jointly vary both *Strike Amplitude* and *Strike Duration* to regulate gesture acceleration were expected to be distributed across both motor coordination strategies, as this combined strategy can effectively scale kinetic energy with task demand, regardless of the specific joint’s amplitude limits.

## Materials and methods

### Participants

Initially, 50 participants took part in the experiment, but only 33 were retained for the final analysis. Seventeen participants were excluded due to problems (missing data due to marker occlusion) related to the reflective marker placed on the tip of the index finger. The position of this marker was essential for determining the moment of contact, but under some conditions, it was not always visible. Finally, the data of 33 right-handed individuals (22 males and 11 females) with normal or corrected vision were analyzed. The average age was 25 years (range: 19 to 43 years). None of them reported any physical injury or pathology that would alter a striking gesture. An a priori power analysis (G*Power 3.1.9.7; Faul et al. 2007) was conducted to determine the sample size for a three-level between-subjects comparison (cf. our hypothesis in the *Introduction*). We estimated a conservative effect size of Cohen’s *f* = 0.60 based on the aforementioned literature comparing expert and novice spatio-temporal parameters in golf putting. Where original reports did not provide Cohen’s *f*, we converted between-subjects ANOVA results into *f* values. These studies reported significant expertise effects on downswing duration (Delay et al. 1997: *f* = 0.52), amplitude (Delay et al. 1997: *f* = 0.43; Sim and Kim 2010: *f* = 1.97), velocity at impact (Delay et al. 1997: *f* = 0.69; Sim and Kim 2010: *f* = 0.91), and coefficient of variation of impact velocity (Hasegawa et al. 2017: *f* = 1.08). While these findings suggest an average expertise-related effect size *f* of approximately 0.93 despite small samples (4 < *n* < 11), we assumed that the planned clustering analysis would also inherently favor between-group differences. Nevertheless, we adopted a more conservative *f* = 0.60 to account for the potential variability in motor strategies among unselected participants performing a novel task (striking an object to a target distance). With a power of at least 80% (alpha = 0.05), a minimum of 30 participants was required; we concluded recruitment at *n* = 33 participants with complete, valid data. A local ethics committee approved the experiment (CER-Paris-Saclay-2018-021-R). The dominant hand was checked with the Edinburgh test (Oldfield 1971) after they read and signed the consent form.

## Experimental setup

**Fig. 1:**
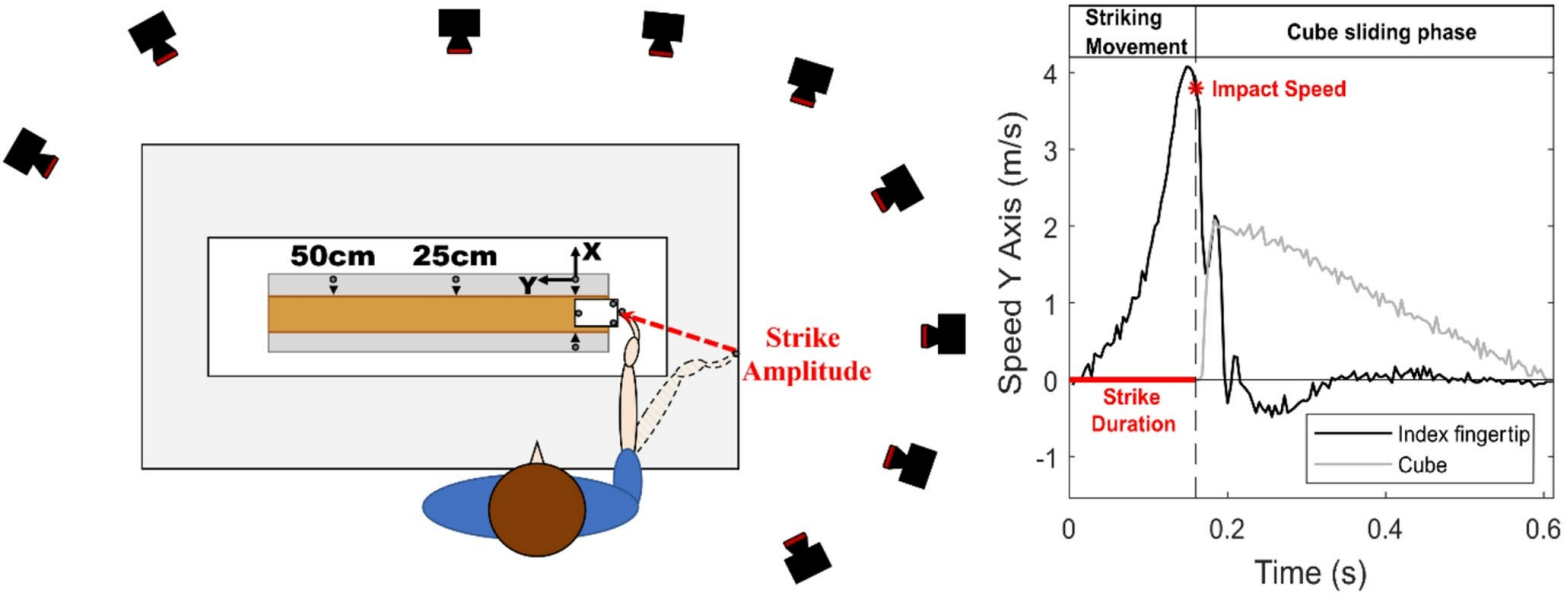
Left panel: Illustration of the experimental setup. The participant should strike a cube (while keeping the elbow at a fixed position on the table), which then slides toward a target distance (25–50 cm) in a gutter. Optoelectronic cameras record the movement of the index finger and the cube relative to the target distance indicated by reflective markers on the gutter. Right panel: Strike Amplitude corresponds to the fingertip displacement over the Strike Duration, from movement onset to Impact Speed. Cube speed increases during strike contact and decreases due to friction until it stops.

The behavioral task consisted of hitting a PLA cube-like object (46 g, length = 60 mm, width and height = 50 mm) so that its front edge stopped at a target distance, as illustrated in Fig. 1. Hereafter, the object will be referred to as “cube”. The cube slides inside a gutter-shaped experimental device placed on a motorized table that allowed adjusting the height of the gutter as a function of the participant, so that the index finger striking the cube was level with the target center. In addition, the gutter inclination could be varied mechanically. More details can be found in Famié et al. (2022).

The lateral sides of the gutter were made of aluminum, and its internal dimensions were 800 mm long, 55 mm wide, and 25 mm high. At the base of the gutter, there was a space to insert the different surfaces used for the experiment, aluminum and balsa wood. The two target distances to be achieved were 25–50 cm, indicated by a triangular sticker and reflective marker on the upper side of the gutter. The motorized table supporting the device allowed the surfaces to be tilted either − 10°, 0°, or + 10°.

For each trial, participants positioned their elbow at a fixed point marked on the table, allowing them to rotate their forearm while maintaining the elbow’s position (Fig. 1). They were instructed to keep their forearm approximately perpendicular to the apparatus initially before moving. Seating adjustments ensured that the arm and forearm formed an approximately right angle with the elbow comfortably resting on the table. During the striking task, participants were free to choose their motor strategy as long as the elbow remained stationary. The objective was to strike the cube so that it slid along the gutter and the front edge stopped at the target distance. The experimenter reset the cube to the starting position after each trial.

The experiment involved 12 conditions, created by combining two target distances (25 cm and 50 cm), two surface types (balsa wood and aluminum), and three surface slopes (-10°, 0°, + 10°). Each participant completed 120 trials, with 10 repetitions for each condition. Participants started the experiment directly, without any preliminary practice trials. The experiment followed a nested design: participants completed 60 trials at 25 cm, followed by 60 trials at 50 cm. Within each distance, they tested both balsa wood and aluminum surfaces in different blocks (*n* = 30 trials each). For each surface, they performed three surface-slope blocks (with 10 consecutive trials per block), always beginning with 0°, followed by a counterbalanced order of + 10° and − 10° slopes. These different block orders resulted in four groups, as detailed in Famié et al. (2022) Table 1 (see 10.1371/journal.pone.0264370.t001).

## Data acquisition and analysis

An OptiTrack™ motion capture system was used during the experiment to record motion at 250 Hz using 9 cameras (model S250e). Reflective markers were placed on the cube (4 mm), the experimental device (7 mm), and the participant’s right arm and hand (4 mm). The present study is a re-analysis of the motion capture data from Famié et al. (2022), focusing on the movement of the index fingertip as measured by a reflective marker on the nail of the index finger. The data that support the findings of this study are available in the Zenodo repository (https://zenodo.org/records/19924581).

Motion capture data was collected using AMASS v2016 software and subsequently processed with custom MATLAB R2020a routines to prepare the files required for statistical analysis in MATLAB, R version 4.4.2 (the R Jupyter Notebooks are available in the Zenodo repository), and JASP 0.95.4. A low-pass filter was not applied to the 3D marker position data because typical filter parameters from human movement studies (e.g., 10–15 Hz cutoff, second or third order Butterworth) would have resulted in the collision event (lasting approximately 15 ms) being treated as noise and thus removed. Instead, we applied a *Multiple Sources of Information* method to detect the impact between the finger and the cube (for details, see Famié et al. 2022). The speed of the index fingertip and the cube was computed using MATLAB’s *diff* function, which applies a first-order forward finite-difference (Euler forward) method. The ‘Multiple Sources of Information’ method (MSI, Schot et al., 2010; Smeets et al., 2010) described in the previous article (Famié et al. 2022) was optimized to detect the frame just before the impact (*t*_contact_), based on the peak of deceleration of the fingertip at impact (Eq. 1).

1 $$\:{t}_{contact}=\:min\left\{t\:\left(\frac{\mathrm{A}\mathrm{c}\mathrm{c}\mathrm{e}\mathrm{l}\mathrm{e}\mathrm{r}\mathrm{a}\mathrm{t}\mathrm{i}\mathrm{o}\mathrm{n}}{\mathrm{M}\mathrm{i}\mathrm{n}\:\mathrm{A}\mathrm{c}\mathrm{c}\mathrm{e}\mathrm{l}\mathrm{e}\mathrm{r}\mathrm{a}\mathrm{t}\mathrm{i}\mathrm{o}\mathrm{n}}\right)>\:0.4\:\right\}-\mathrm{d}t$$

The analysis focused on the time window bounded by the instant of maximal index fingertip speed (*t*_maxFS_) and the moment of minimal cube speed (*t*_minCS_) immediately preceding the cube’s motion. The cube motion threshold was defined as cube velocity superior at 0.04 m/s. Within this interval, the acceleration of the index fingertip was normalized by dividing by the minimum acceleration observed during this time interval [*t*_maxFS_; *t*_minCS_]. The time of contact was then defined as the last instant (-d*t*) preceding a normalized fingertip deceleration superior to 40% of this minimum value (0.4 in Eq. 1). This threshold-based criterion was adopted to ensure robust detection of time contact because, in some cases, the index fingertip was already in contact with the cube and had started to decelerate before the cube began to move.

The combination of the different experimental factors, surface material (aluminum, balsa wood), surface slope (-10°, 0°, + 10°), and target distance (25 cm, 50 cm), created a continuum of task demand, which we quantified using an optimal cube speed required to reach the target distance (*d*). *Optimal Cube Speed* was computed on the basis of Eq. 2:

2 $$\:Optimal\:Cube\:Speed=\:\sqrt{2\:g\:d\:\left[sin\left(\alpha\:\right)\:+{\mu\:}_{K}\:cos\left(\alpha\:\right)\right]}$$

**Fig. 2:**
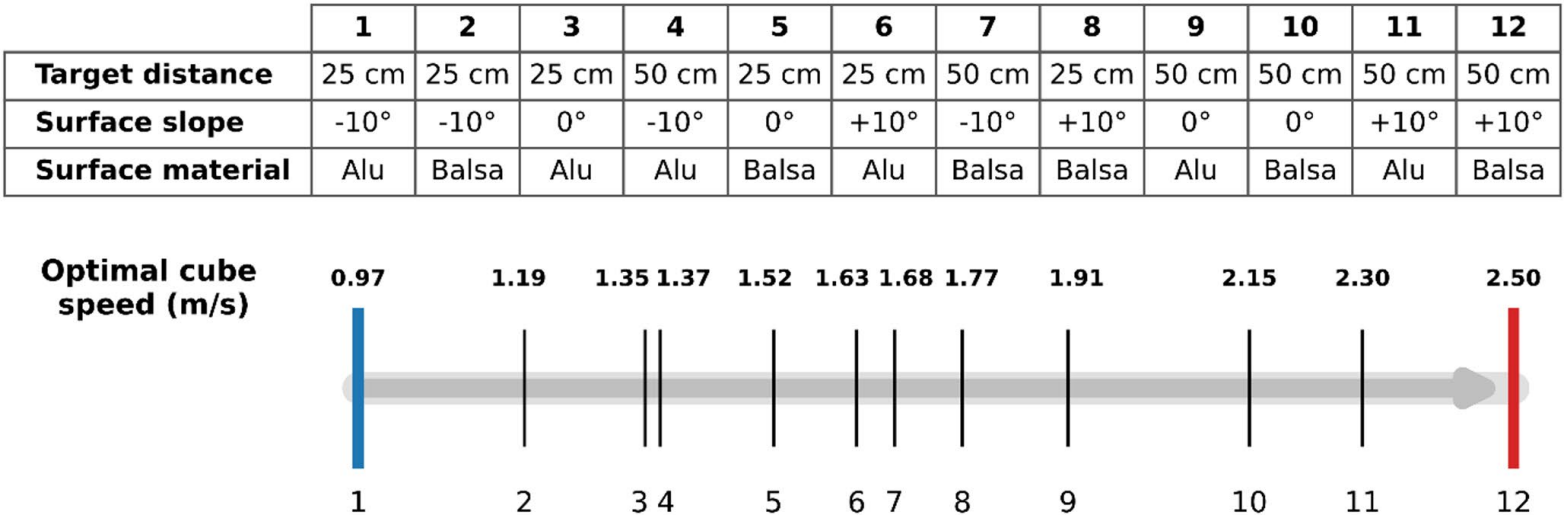
Optimal Cube Speed scale (m/s) representing task demand across experimental conditions (from condition 1 to 12)

The kinematic coefficients of friction were characterized in Famié et al. (2022) for the two surface materials: aluminum ($\:{\mu\:}_{K}$= 0.37) and balsa wood ($\:{\mu\:}_{K}$= 0.47). The deceleration of the object is influenced by gravity (*g*), the surface slope (α), and the kinematic coefficients of friction ($\:{\mu\:}_{K}$). *Optimal Cube Speed* was calculated for each condition along a task-demand continuum (Fig. 2) from 0.97 m/s (aluminum, -10°, 25 cm) to 2.50 m/s (balsa, + 10°, 50 cm).

## Performance variables

The strike ballistic movement was segmented in 3 phases: the *arming phase* when the index finger goes away from the cube; the *strike phase* which finishes just before the contact between the index finger and the cube; and the *cube sliding phase* which started when the cube is set into motion (for an illustration, see Famié et al. (2022): 10.1371/journal.pone.0264370.g002). In the present study, we analyzed the spatio-temporal regulation of the striking phase by focusing on the control of the following parameters: (a) *Strike Duration* (between the moment the strike is initiated and the moment the tip of the index finger comes into contact with the object corresponding to the point of maximum deceleration of the tip of the index finger upon impact, see Eq. 1), (b) *Strike Amplitude* (distance between the position of the tip of the index finger when the striking motion is initiated and its position when the tip of the finger reaches its maximum deceleration point upon impact), and (c) *Impact Speed* (speed of the index finger tip at impact with the cube). Finally, we also examined the spatial error (the distance between the cube’s front edge and the target distance) as a percentage of the target distance.

### Statistical analysis

For each participant, Pearson’s *r* correlations between three parameters of interest were calculated based on performance at 120 trials, namely between: *Strike Duration* and *Impact Speed*,* r*(*D*,* IS*); *Strike Duration* and *Strike Amplitude*,* r*(*D*,* A*); and *Strike Amplitude* and *Impact Speed*,* r*(*A*,* IS*). We first identified three distinct modes of regulation among the participants, using r > |0.18| as the criterion, since this corresponds to *p* < 0.05 for *n* = 120 trials. Then, in order to statistically confirm the existence of these three modes of regulation, we performed a K-means clustering analysis, using Hartigan & Wong’s (1979) algorithm, on the *r*-values of the table once converted into Fisher Z-values with the following Equation:

3 $$ Z = {\text{ }}0.{\mathrm{5}}\left[ {{\mathrm{ln}}\left( {{\mathrm{1}} + r} \right){\text{ }}{-}{\text{ ln}}\left( {{\mathrm{1}} - r} \right)} \right] $$

The optimal number of clusters was determined on the basis of both the Elbow method and the two-dimensional t-Distributed Stochastic Neighbor Embedding (t-SNE) plot.

Then, we performed a kinematic analysis of each spatio-temporal strategy for strike control. The analysis of striking behavior was conducted at two complementary levels: the strength of kinematic coupling within individual trials and the magnitude of parameter scaling across varying task demands. First, the Pearson correlation coefficients *r*(*A*, *D*), *r*(*IS*, *A*), and *r*(*IS*, *D*) were used to quantify the coupling strength, reflecting the degree of interdependence between spatial and temporal parameters and their respective influences on the final velocity for each representative participant. While these correlations describe the consistency of the motor control strategy, they do not quantify the rate of change. Therefore, we subsequently analyzed the slopes of the linear regressions for *Strike Amplitude* (*A*), *Strike Duration* (*D*), and *Impact Speed* (*IS*) as a function of *Optimal Cube Speed*. By using *Z*-scored slopes, we normalized the disparate units of meters and seconds, enabling a quantitative comparison of the relative scaling magnitudes of each parameter. This dual approach reveals how individual trial-by-trial consistency translates into the distinct, group-level movement strategies observed across the clusters. Finally, to compare individual mean slope values across clusters, we performed an ANOVA with Cluster as a between-subjects factor. For each representative participant per cluster, we illustrate the speed and position profile of the finger movement for the 10 trials for the less (25 cm alu − 10° in blue) and most (50 cm balsa + 10° in red) demanding conditions, and then the scatterplot of our previously mentioned correlations of interest for all the trials of the participant. Finally, we applied Hierarchical Multiple Factor Analysis (HMFA, see Pagès, 2014), an extension of Multiple Factor Analysis (MFA, see Abdi et al. 2013), using Cluster as a categorical variable and three data blocks of continuous variables: *Strike Amplitude*, *Strike Duration*, and *Impact Speed*. Within each block, performance across the 12 levels of task demand was treated as a repeated-measures factor. This method of analysis balances the roles of the groups of variables at each level of the hierarchy and provides outputs that may be interpretable from an overall perspective (the hierarchical structure) as well as from various perspectives at the various levels of the hierarchy (Dien and Pagès 2003). HMFA was conducted using the FactoMineR R package (Lê et al. 2008), and the factoextra R package was used to visualize the HMFA results. The R code (Jupyter Notebook) and output results are available in Zenodo ( https://zenodo.org/records/19924581).

More precisely, HMFA handles mixed data by placing all variable groups on a comparable footing before combining them into a global analysis. Each block of variables is first processed using the appropriate factorial method for its data type: Principal Component Analysis (PCA) for continuous variables and Multiple Correspondence Analysis (MCA) for categorical variables (which is mathematically a PCA performed on a complete disjunctive table; Lê et al. 2008). This step transforms all blocks (regardless of data type) into numerical coordinate systems, allowing individuals, variables, and category levels to be represented together. To ensure balanced influence, HMFA applies a variance-based weighting: every block is divided by the square root of its first eigenvalue (1/√λ₁), which standardizes the maximum variance each block can contribute. This guarantees that no block dominates solely because it contains more variables or stronger internal correlations. Once transformed and weighted, all blocks are merged, and a single global PCA is performed. The resulting common dimensions summarize the strongest shared structure across all blocks and hierarchical levels. Importantly, these dimensions also allow meaningful comparison of contributions: each block, variable, category, or individual contributes a quantifiable proportion of the explained variance (inertia). This makes it possible to determine which parts of the hierarchical structure of the data blocks, from groups and subgroups to individual variables, specific categories, or observations, contribute most to shaping each global dimension. Because this architecture depends on the original, unrotated eigenstructure and on the propagation of standardized variances through the hierarchy, factor rotations such as Varimax cannot be applied. Rotation would redistribute variance across axes, break the λ₁-based weighting scheme, distort contributions, and invalidate the HMFA compromise space.

## Results

### Correlational and cluster analysis of interindividual differences in the spatio-temporal regulation of striking movement

Table 1 illustrates the individual *r* values ranked in ascending order of correlation between *Strike Duration* and *Impact Speed* (see second column). Although all participants show a positive and significant correlation between *Strike Amplitude and Impact Speed*,* r(A*,* IS)*, visual inspection of the data indicates that the correlation between *Strike Duration* and *Impact Speed*,* r*(*D*,* IS*), shows greater interindividual variability, ranging from − 0.60 to 0.44. More precisely, we identified three distinct modes of regulation adopted by the participants using *r* > |0.18| as the criterion (see *Statistical analysis* section). As a consequence, the first regulation mode (11 participants) would correspond to significant negative correlation values (*r* < -0.18) for *r*(*D*,* IS*), suggesting a temporal regulation of the movement in which decreasing the *Strike Duration* is used to increase *Impact Speed*. The second regulation mode (11 other participants) shows non-significant correlations (-0.18 < *r* < 0.18) for *r*(*D*,* IS*), suggesting that regulation is based on the *Strike Amplitude* parameter significantly positively correlated with the other two parameters. Finally, for the third regulation mode (11 other participants), participants show significant positive correlations (*r* > 0.18) for *r*(*D*,* IS*), and between each other’s parameters, suggesting a modulation of the acceleration of the movement before impact (as discussed later on).

**Table 1:** Individual data (*n* = 120 trials per participant) on correlations (*r*) between each parameter of interest for the 33 participants retained for the analysis, who did not show any data recording problems among the 50 initial participants (hence the numbering). Significant correlations (*p* < 0.05), corresponding to *r* > |0.18|, are indicated with a star. The dashed lines separate each of the three clusters

Participant	Strike Duration & Impact Speed	Strike Duration & Strike Amplitude	Impact Speed & Strike Amplitude
29	−0.60 *	0.24 *	0.31 *
50	−0.54 *	0.24 *	0.41 *
37	−0.53 *	−0.11	0.75 *
41	−0.50 *	0.31 *	0.52 *
27	−0.46 *	0.67 *	0.17
14	−0.45 *	0.29 *	0.48 *
16	−0.36 *	0.38 *	0.60 *
10	−0.36 *	0.46 *	0.54 *
20	−0.36 *	0.04	0.64 *
19	−0.34 *	-0.02	0.81 *
44	−0.34 *	0.22 *	0.71 *
26	−0.12	0.46 *	0.64 *
38	−0.09	0.24 *	0.80 *
17	−0.08	0.34 *	0.74 *
33	−0.03	0.66 *	0.58 *
21	−0.02	0.52 *	0.48 *
32	0.04	0.47 *	0.67 *
42	0.05	0.47 *	0.77 *
46	0.07	0.31 *	0.85 *
30	0.08	0.53 *	0.74 *
13	0.10	0.51 *	0.80 *
45	0.11	0.59 *	0.70 *
11	0.19 *	0.66 *	0.68 *
31	0.27 *	0.75 *	0.71 *
39	0.27 *	0.74 *	0.72 *
43	0.27 *	0.80 *	0.66 *
40	0.28 *	0.88 *	0.57 *
25	0.29 *	0.64 *	0.71 *
18	0.32 *	0.81 *	0.66 *
36	0.38 *	0.75 *	0.76 *
12	0.39 *	0.65 *	0.88 *
35	0.40 *	0.75 *	0.79 *
24	0.44 *	0.57 *	0.81 *

**Fig. 3:**
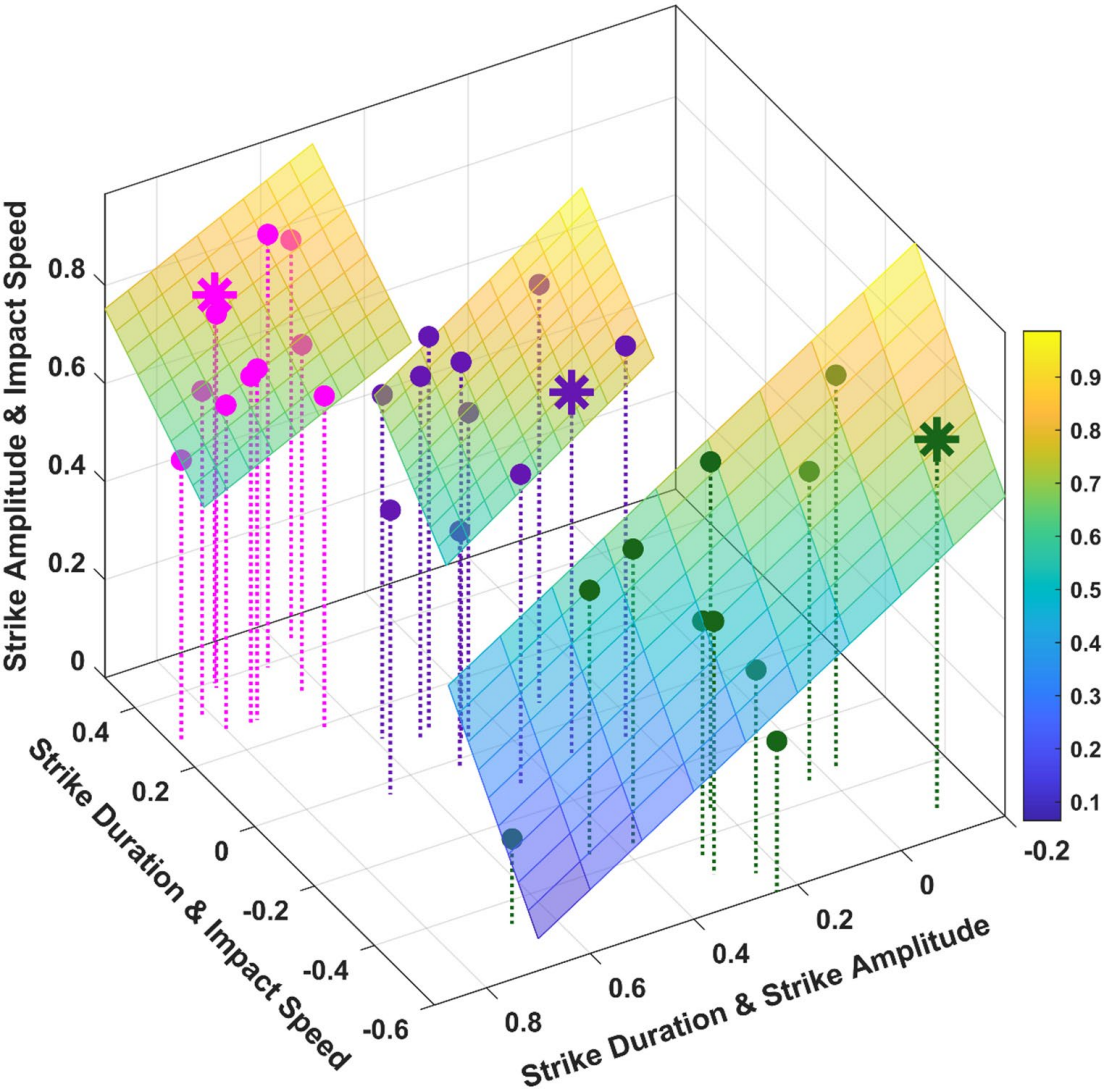
This 3D plot illustrates the individual correlation data for each cluster. In green, the Cluster 1 (“Inverse Duration-Amplitude Strategy”). In purple, the Cluster 2 (“Temporal Invariance Strategy”). In magenta, the Cluster 3 (“Late-Impulse Strategy”). Furthermore, for each cluster, participants used as examples in the main text are indicated by a star point

Three clusters of 11 participants each, were extracted corresponding to the 9 cells of Table 1 and the data point clouds illustrated in Fig. 3. Along the lines of our previous description of Table 1, as well as Fig. 3, we labeled Cluster 1 as “*Inverse Duration-Amplitude Strategy*” (for participants who showed significant negative *r*(*D*,* IS*) values, see green dots); Cluster 2 as “*Temporal Invariance Strategy*” (for the participants who showed nonsignificant *r*(*D*,* IS*) values, see purple dots); Cluster 3 “*Late-Impulse Strategy*” (for participants who showed significant positive *r*(*D*,* IS*) values, see magenta dots).

**Fig. 4:**
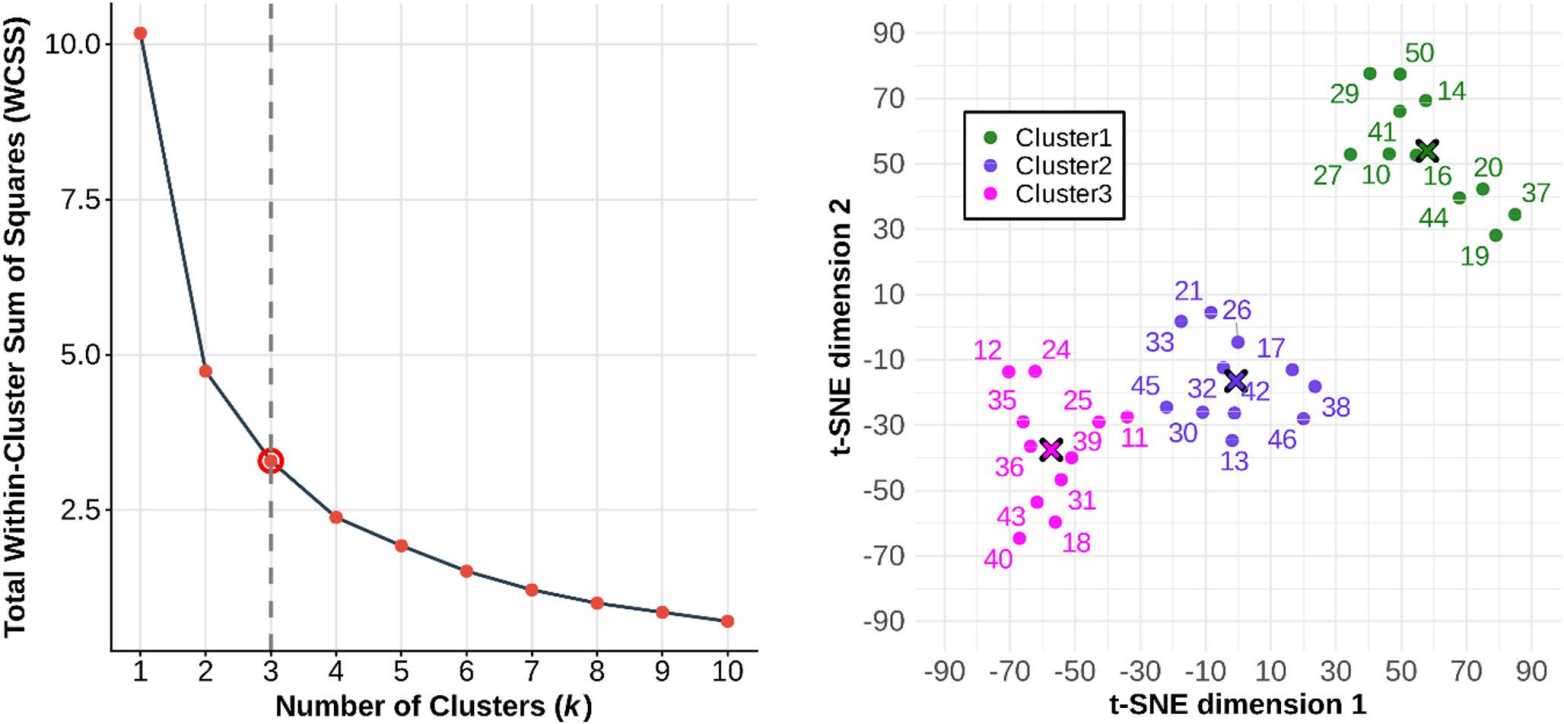
Cluster validation and strategy classification. Left panel: Elbow plot demonstrating the reduction in WCSS across cluster solutions; the inflection point at k = 3 indicates the optimal balance between model complexity and variance explanation. Right panel: t-Distributed Stochastic Neighbor Embedding (t-SNE) visualization of the three-cluster solution. Centroids for each strategy are highlighted with an **‘X’**

The three-cluster solution was selected based on the elbow method (see Fig. 4, left panel) and the analysis of marginal variance explanation. A primary structural split was observed at *k* = 2, accounting for 53.5% of the total variance. Increasing the complexity to *k* = 3 provided a further 14.2% improvement, identifying a distinct third coordination strategy. Beyond this point, the model exhibited clear diminishing returns; the transition to *k* = 4 and *k* = 5 yielded only 8.9% and 4.5% improvements, respectively. The inflection point at *k* = 3 thus represents the optimal trade-off between model parsimony and explanatory power, accounting for 67.7% of the total variance in the Table 1 data (between-cluster sum of squares/total sum of squares = 0.677). Moreover, the Calinski-Harabasz index (Calinski and Harabasz 1974) confirmed a robust group structure, *F*(2, 30) = 31.41, *p* < 0.0001, indicating that the differences between the three spatio-temporal strategies are significantly greater than the individual variation within those strategies. The total within-cluster sum of squares (WCSS) was 3.46, distributed across the groups as follows: Cluster 1 = 1.59, Cluster 2 = 0.91, and Cluster 3 = 0.78. These values indicate that, while all clusters are statistically distinct, Cluster 1 exhibits greater internal variability in the correlation between performance variables than the more cohesive strategies observed in Clusters 2 and 3.

To visualize the high-dimensional structure of the data, a nonlinear dimensionality reduction was performed using t-Distributed Stochastic Neighbor Embedding (t-SNE; Van Der Maaten 2014). The t-SNE visualization (Fig. 4, right panel) yielded three discrete clusters with no evidence of bridge observations (except maybe for participant 11). This topological separation confirms that the *k* = 3 solution identifies distinct spatio-temporal strategies for regulating the striking movement rather than arbitrary partitions of a continuous distribution.

## Kinematic description of each spatio-temporal strategy of the strike control

**Fig. 5:**
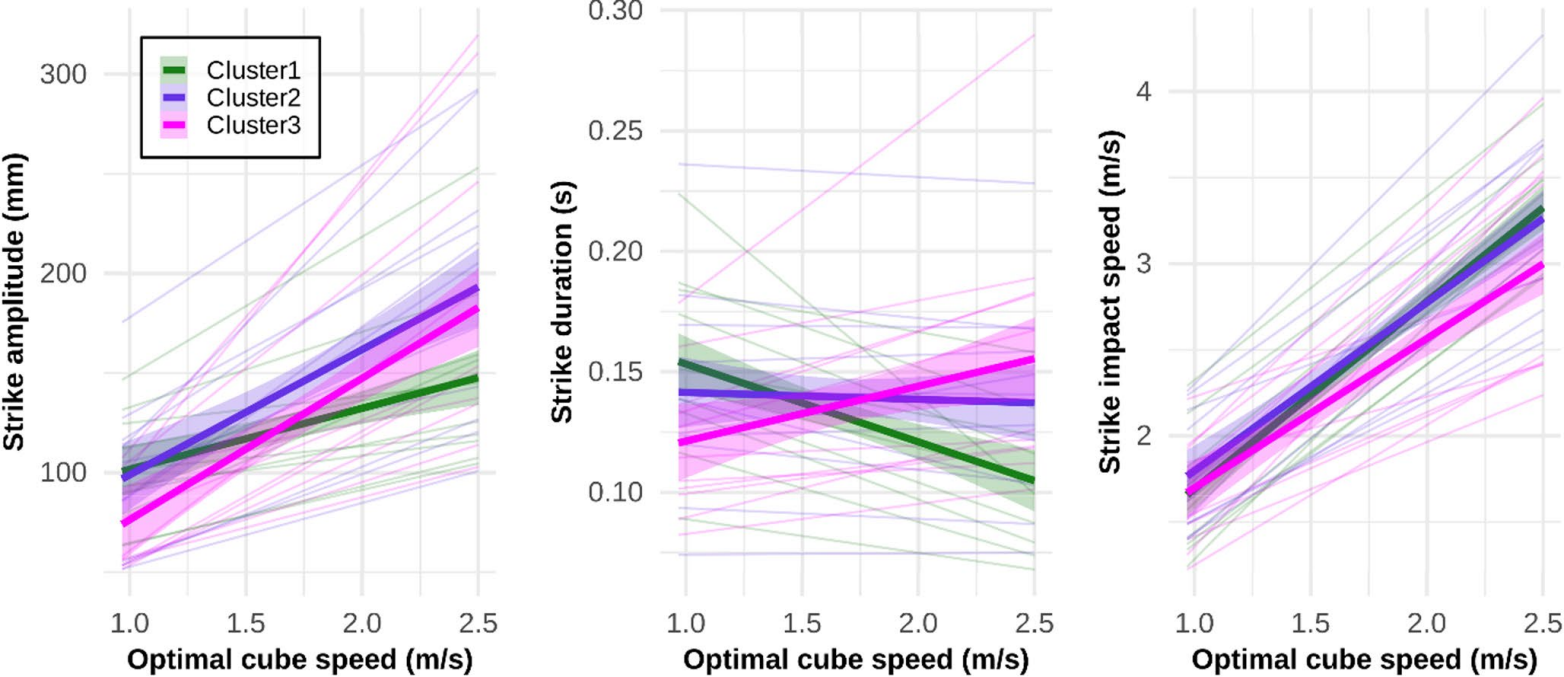
Modulation of each variable of interest as a function of task demand, quantified as an Optimal cube speed for the strike. The mean linear fit for each cluster is shown as a thick line (with a 95% CI ribbon), while individual fits are shown as thinner lines

We first examined, at the cluster-group level, how different variables of interest were modulated by task difficulty, quantified as optimal strike speed, using linear regression. As illustrated in Fig. 5 right panel, the strike *Impact Speed* increased with task demand as required. An ANOVA on the individual slopes shows that no significant difference between clusters, *F*(2, 30) = 1.72, *p* = 0.20, $\:{\eta\:}_{p}^{2}$ = 0.11, 95% CI [0, 0.31], confirming equivalent levels of performance across clusters. Moreover, the mean slopes of all clusters did not differ significantly from 1 (Cluster 1: *M* = 1.09, 95% CI [0.92, 1.26]; Cluster 2: *M* = 0.98, 95% CI [0.80, 1.15]; Cluster 3: *M* = 0.87, 95% CI [0.69, 1.04]), as indicated by the 95% confidence intervals encompassing the value 1. Where the clusters differ is in how *Strike Amplitude* and *Strike Duration* are modulated by task demand, as shown in the left and middle panels of Fig. 5, respectively. *Strike Amplitude* slopes differed significantly between clusters, *F*(2, 30) = 5.74, *p* = 0.008, $\:{\eta\:}_{p}^{2}$ = 0.28, 95% CI [0.03, 0.49]. Tukey post-hoc comparisons showed that the slope for Cluster 1 (*M* = 30.71, 95% CI [12.52, 48.90]) was significantly shallower than for Cluster 2 (*M* = 62.89, 95% CI [44.70, 81.07]), *p* = 0.041, Cohen’s *d* = 1.09, 95% CI [−0.05, 2.23], and than for Cluster 3 (*M* = 71.05, 95% CI [52.86, 89.24]), *p* = 0.009, Cohen’s *d* = 1.37, 95% CI [0.20, 2.54]. Cluster 2 and 3 slopes did not differ significantly, *p* = 0.79, Cohen’s *d* = 0.28, 95% CI [-0.81, 1.36]. In each cluster, the 95% CI for the *Strike Amplitude* slope was entirely above zero, indicating that all slopes were significantly positive. Finally, *Strike Duration* slopes differed significantly between clusters, *F*(2, 30) = 31.79, *p* < 0.0001, $\:{\eta\:}_{p}^{2}$ = 0.68, 95% CI [0.46, 0.79]. Tukey post-hoc comparisons showed that all slopes differed significantly from one another, *p*s < 0.003, Cohen’s *d*s > 1.57. The slope for Cluster 1 (*M* = -0.032, 95% CI [-0.042, -0.022]) was significantly negative, whereas the slope for Cluster 3 (*M* = 0.023, 95% CI [0.013, 0.033]) was significantly positive, and that for Cluster 2 (*M* = −0.003, 95% CI [−0.013, 0.007]) did not differ from zero (see 95% CIs).

**Fig. 6:**
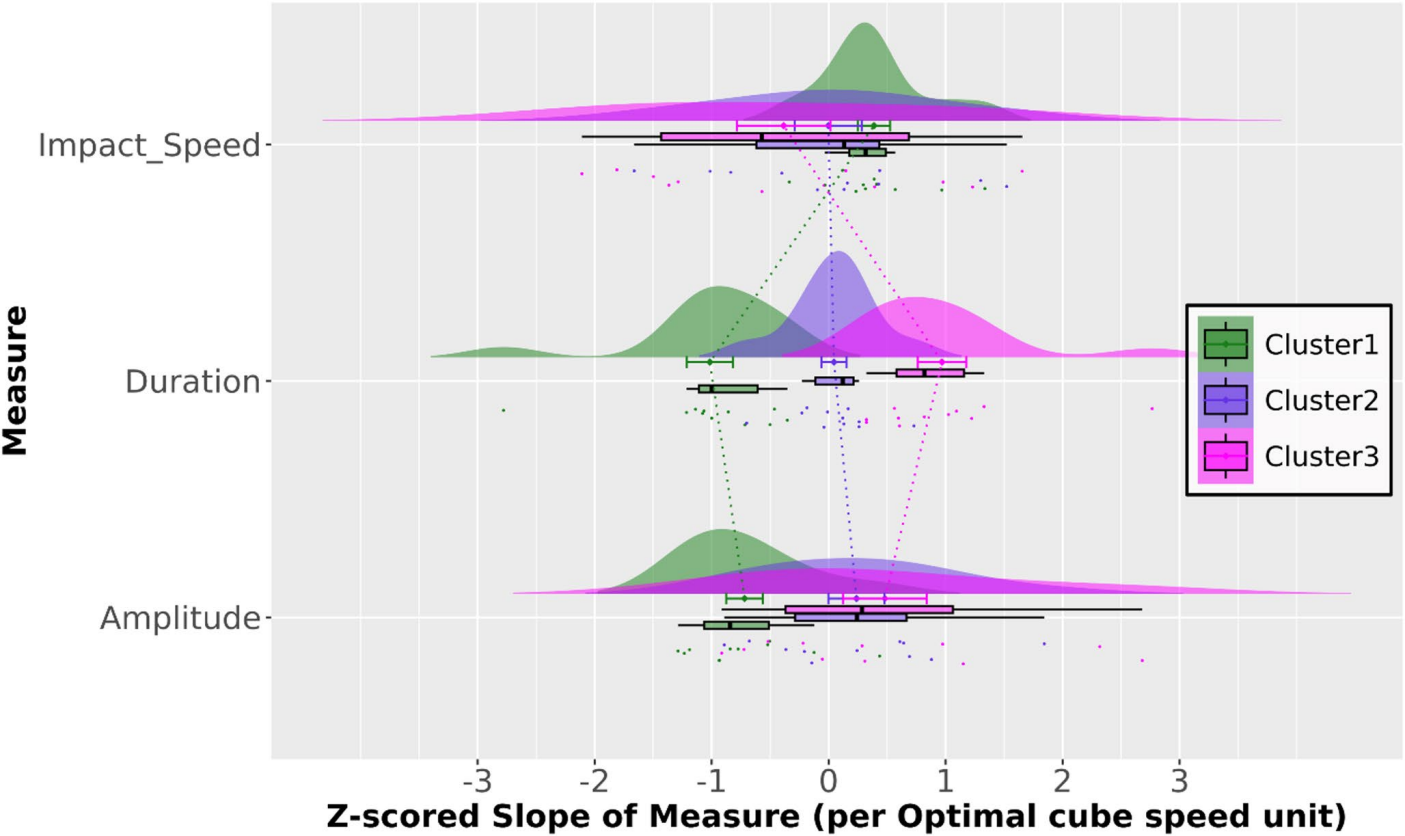
Rainclouds (Allen et al. 2021) of the Z-scores for the slopes illustrated in Fig. 5

To compare spatial and temporal scaling directly, we converted the raw regression coefficients (*Raw*_Slope_) into *Z*-scored slopes (*Z*_Slope_). This normalization accounts for the different units (meters for the spatial scaling of *A* and seconds for the temporal scaling of *D*) allowing us to determine which factor more strongly influences the final *Impact Speed* within each cluster. The distributions shown in Fig. 6 reflect the interindividual variability within each cluster. Analysis of normalized and raw slope coefficients, derived from the linear regression of movement parameters against *Optimal Cube Speed*, confirmed that participants exhibited three distinct strike control strategies. The behaviors of the three representative participants (37, 17, 35) are presented below with specific reference to their group’s calculated parameters to illustrate the distinct characteristic features of each cluster.

Cluster 1 “*Inverse Duration-Amplitude Strategy*” is defined by its structural reliance on temporal compression of the movement. The scaling behavior for this policy is characterized by the most extreme negative *Z*-scored slope for *Strike Duration* (*D*) across clusters (*Z*_Slope_(*D*) = −1.01). This metric indicates that Cluster 1 exhibited the highest relative rate of *D* reduction. Participant 37 exemplified this behavior with a *Raw*_Slope_(*D*) = -0.034 s / (m/s). While the raw slope for *Strike Amplitude* remains positive, indicating an absolute increase in movement range, the relative modulation of *A* for Cluster 1 was the lowest across clusters (*Z*_Slope_(*A*) = -0.72). This kinematic profile resulted in the highest *Raw*_Slope_(*IS*) across all three strategies (+ 1.09), quantifying the greatest rate of final velocity increase for this group (*Z*_Slope_(*IS*) ≈ + 0.39).

Cluster 2 “*Temporal Invariance Strategy*” is characterized by *Strike Duration* invariance. The *Strike Duration* scaling for this cluster was near zero, with *Z*_Slope_(*D*) = + 0.05. Participant 17’s *Raw*_Slope_(*D*) = -0.0005 s / (m/s) demonstrates this invariant timing structure. Speed modulation in this cluster was achieved almost exclusively through spatial scaling (*Z*_Slope_(*A*) = + 0.24).

Finally, Cluster 3, “*Late-Impulse Strategy*”, is defined by the synchronous expansion of both spatial and temporal parameters of the strike movement. This cluster was uniquely defined by the highest positive *Z*-scored slope for *Strike Duration* (*Z*_Slope_(*D*) = + 0.97), coupled with a significant absolute increase in movement range. This strategy is exemplified by Participant 35, who demonstrated a *Z*_Slope_(*D*) = + 0.0228 s and a *Z*_Slope_(*A*) = + 71.50 mm. Despite increases in both movement parameters, the Cluster 3 strategy resulted in the lower rate of final velocity increase (*Raw*_Slope_(*IS*) = + 0.87) across clusters, further confirmed by a negative *Z*-scored slope for *Impact Speed* (*Z*_Slope_(*IS*) = −0.39).

**Fig. 7:**
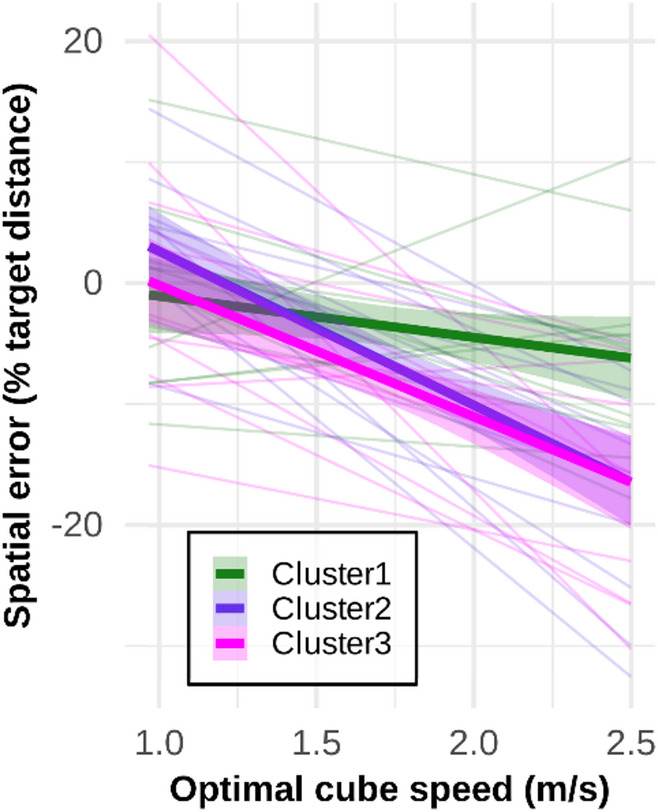
Modulation of spatial error (in percentage of target distance) as a function of task demand. The mean linear fit for each cluster is shown as a thick line (with a 95% CI ribbon), while individual fits are shown as thinner lines

Finally, an ANOVA on spatial error (in percentage of target distance) showed no difference between groups, *F*(2, 30) = 1.164, *p* = 0.326, (*M*_Cluster1_= −3.44%, 95% CI [−7.54, 0.65]; *M*_Cluster2_ = -6.19%, 95% CI [−10.28, −2.01]; *M*_Cluster3_ = −7.71%, 95% CI [−11.8, −3.62]), $\:{\eta\:}_{p}^{2}$ = 0.07, 95% CI [0, 0.26]. In addition, to examine the effect of task demand on performance, we conducted a regression analysis illustrated in Fig. 7, followed by an ANOVA on the individual slopes for each cluster. The ANOVA showed that the effect of task demand on the regression slopes of spatial error varied significantly between clusters, *F*(2, 30) = 5.56, *p* = 0.009, $\:{\eta\:}_{p}^{2}$ = 0.27, 95% CI [0.02, 0.49]. Although Cluster 1 mean slope did not differ significantly from 0, as indicated by the 95% confidence interval encompassing the value 0 (*M* = -3.41, 95% CI [−7.67, 0.86]), those for Cluster 2 (*M* = −12.71, 95% CI [−16.98, −8.45]) and Cluster 3 (*M* = -10.85, 95% CI [−15.11, −6.59]), did. However, although the slopes for Cluster 2 and Cluster 3 differed significantly from that of Cluster 1 (with *p* = 0.01, Cohen’s *d* = 1.34, 95% CI [0.18, 2.51], and *p* = 0.044, Cohen’s *d* = 1.07, 95% CI [−0.06, 2.21], respectively), the two slopes did not differ between each other (*p* = 0.80, Cohen’s *d* = 0.27, 95% CI [−0.82, 1.35]).

**Fig. 8:**
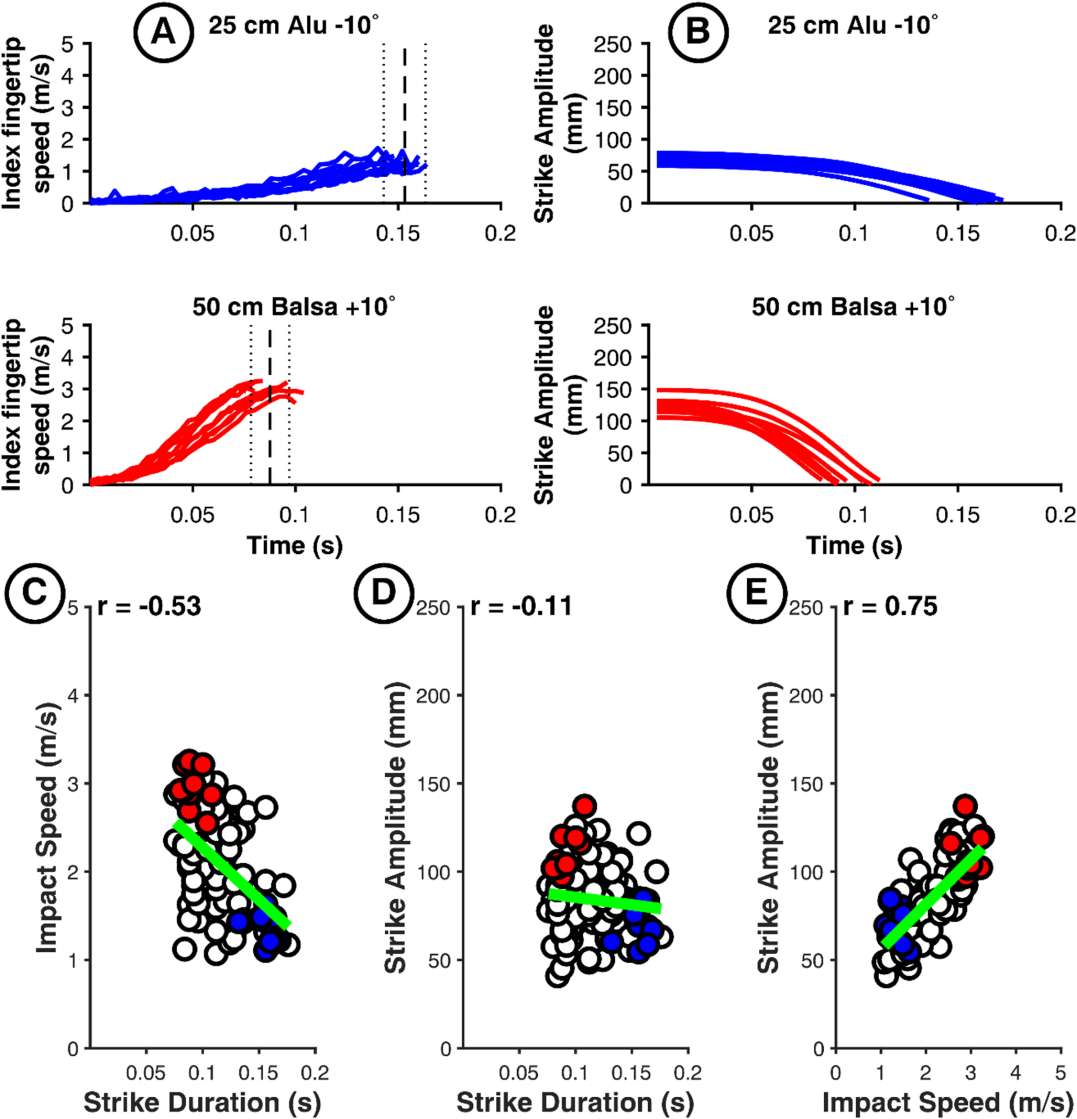
Illustration of Cluster 1 “Inverse Duration-Amplitude Strategy” with the results of participant 37 as an example. Plots of the fingertip speed profiles (A) and Strike Amplitude profile (B) for the 10 trials in the less demanding (blue curves) and most demanding (red curves) conditions. C, D, E: scatterplots for the entire data set (120 trials) of the same participant with the color-coded trials of panel A, for our three variables of interest

To further understand the control strategies of each cluster, we illustrate each strategy using a representative participant from each cluster (indicated by a star in Fig. 3). For example, the kinematic data for Participant 37 (Fig. 8) illustrates an *Inverse Duration-Amplitude Strategy* for scaling movement with task demand (Cluster 1). Higher impact speeds were achieved by shifting the velocity peak earlier in the trajectory (Panel A), effectively reducing *Strike Duration* (*r*(*IS*, *D*) = -0.53, 95% CI [−0.65, −0.38]; Panel C) while simultaneously increasing *Strike Amplitude* (*r*(*IS*, *A*) = 0.75, 95% CI [0.67, 0.82]; Panel E). While *Strike Amplitude* nearly doubled, starting positions were characterized by low inter-trial variability (Panel B). Interestingly, the spatio-temporal coupling between amplitude and duration was weak and nonsignificant (*r*(*A*, *D*) = -0.11, 95% CI [−0.29, 0.07]; Panel D). While several participants in this cluster (including 37, 19, and 20) showed nonsignificant correlations between amplitude and duration (see Table 1), the majority exhibited a significant positive correlation *r*(*A*, *D*). Despite these differences in the *A*-*D* relationship, all participants in Cluster 1 shared the same fundamental pattern of concurrently reducing duration and increasing amplitude to scale impact speed as task demand increased from the “25 cm Alu − 10°” to the “50 cm Balsa + 10°” condition.

**Fig. 9:**
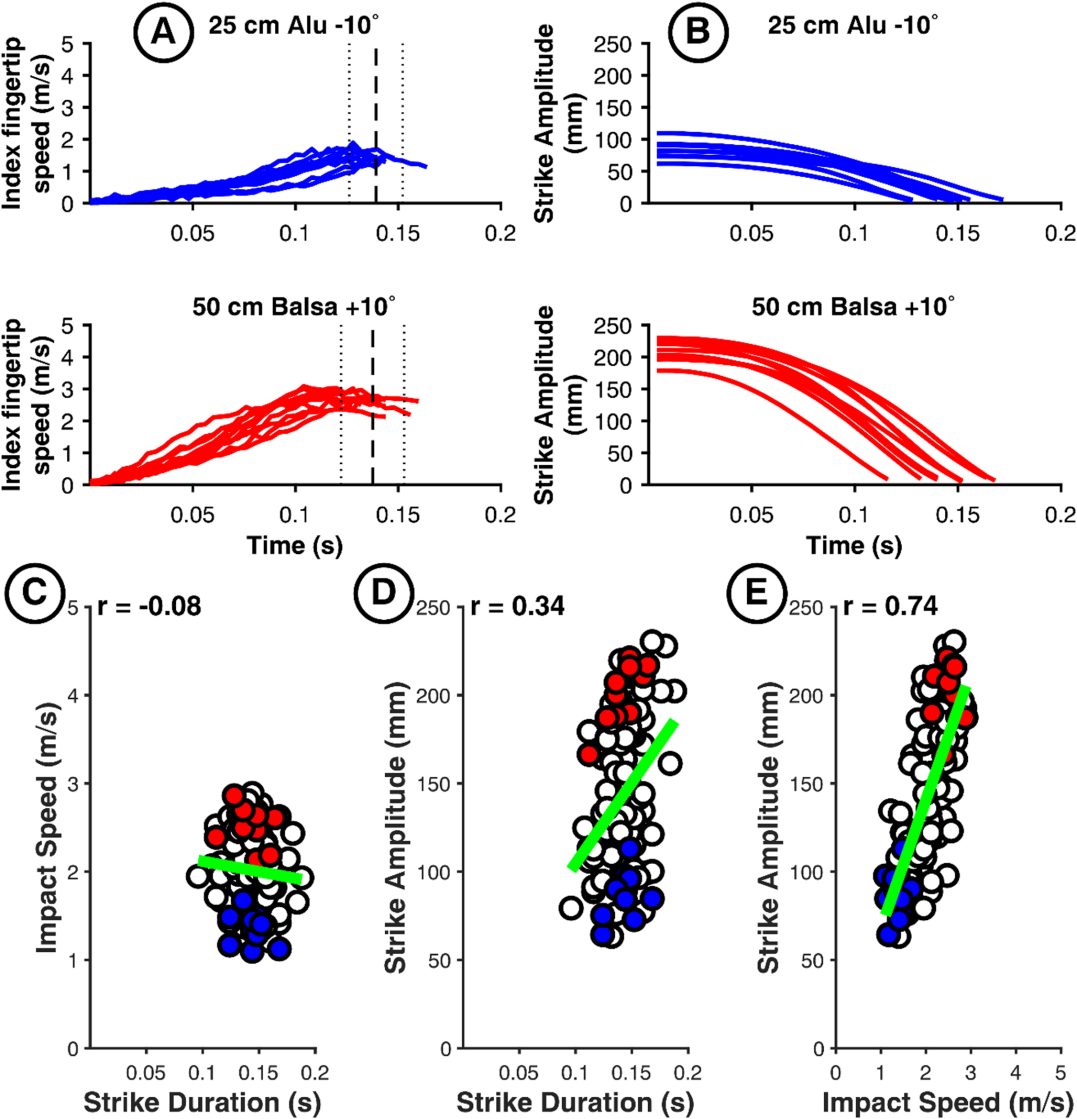
Illustration of Cluster 2 “Temporal Invariance Strategy” with the results of participant 17 as an example. Plots of the fingertip speed profiles (A) and Strike Amplitude profile (B) for the 10 trials in the less demanding (blue curves) and most demanding (red curves) conditions. C, D, E: scatterplots for the entire data set (120 trials) of the same participant with the color-coded trials of panel A, for our three variables of interest

The kinematic data of Participant 17 (Fig. 9) is illustrative of a *Temporal Invariance Strategy* (Cluster 2). The core mechanism for adapting to increasing task demands centers on achieving temporal invariance, thereby minimizing reliance on duration modulation for speed control. Velocity profiles (Panel A) demonstrate this invariant timing, with the movement termination (dashed vertical line) remaining stable across different task difficulties. Instead of reducing movement duration, Participant 17 achieved higher impact speeds by significantly increasing *Strike Amplitude* (up to 225 mm, nearly double that of Participant 37), effectively utilizing a larger spatial window to accelerate the finger to the required velocity while *Strike Duration* remains relatively invariant.

In contrast to Cluster 1, kinematic analysis for Participant 17 reveals a decoupling of impact speed and temporal execution, evidenced by a near-zero correlation between *Impact Speed* and *Strike Duration* (*r*(*IS*, *D*) = -0.08, 95% CI [-0.26, 0.1]; Panel B). While a moderate positive correlation exists between *Strike Amplitude* and *Strike Duration*, *r*(*A*, *D*) = 0.34, 95% CI [0.17, 0.45] (Panel C), *Strike Amplitude* modulation appears to function largely independently of strict temporal scaling. The required speed to comply with task demand is instead modulated via strong spatial coupling, as suggested by the high correlation between *Impact Speed* and *Strike Amplitude* (*r(IS*, *A*) = 0.74, 95% CI [0.65, 0.81]; Panel D). Collectively, these data confirm a control mode characterized by the scaling of spatial magnitude while maintaining temporal invariance.

**Fig. 10:**
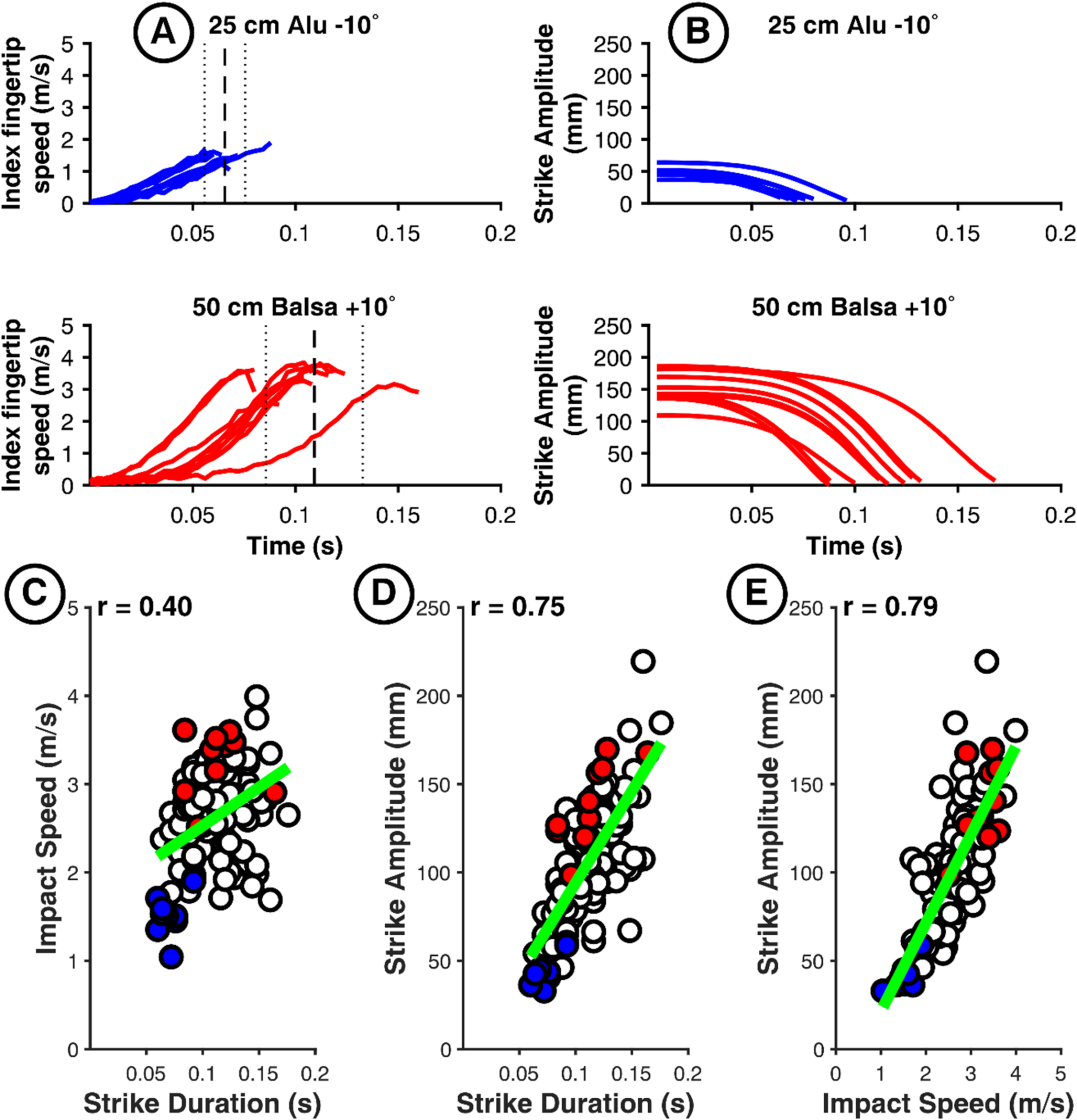
Illustration of Cluster 3 “Late-Impulse Strategy” with the results of participant 35 as an example. Plots of the fingertip speed profiles (A) and Strike Amplitude profile (B) for the 10 trials in the less demanding (blue curves) and most demanding (red curves) conditions. C, D, E: scatterplots for the entire data set (120 trials) of the same participant with the color-coded trials of panel A, for our three variables of interest

Finally, Participant 35 (Fig. 10) illustrates a *Late-Impulse Strategy* (Cluster 3), where adaptation to task demands involves synchronous scaling of spatial and temporal parameters. Rather than a uniform increase in speed, participants utilizing this strategy employ a longer, more controlled approach phase to generate a sharp, late-stage impulse. Velocity profiles (Panel A) show that while the “25 cm Alu − 10°” condition (blue) is near-linear, the “50 cm Balsa + 10°” condition (red) displays lower initial acceleration followed by a steeper terminal increase. This rightward shift is marked by a wide temporal spread and a nearly threefold increase in *Strike Amplitude* variability, suggesting that the increased task demand challenged the participant’s ability to maintain a stable motor program.

This strategy reverses the standard speed-duration trade-off, showing a positive correlation between *Impact Speed* and *Strike Duration* (*r*(*IS*, *D*) = 0.40, 95% CI [0.23, 0.54]; Panel C). The integrated scaling of the motor program is confirmed by strong positive couplings between *Impact Speed* and *Strike Amplitude* (*r*(*IS*, *A*) = 0.79, 95% CI [0.71, 0.85]; Panel E) and between *Strike Amplitude* and *Strike Duration* (*r*(*A*, *D*) = 0.75, 95% CI [0.66, 0.82]; Panel D). These correlations indicate that temporal stretching facilitates a longer preparatory phase, followed by a steep terminal acceleration to satisfy velocity requirements.

### Hierarchical multiple factor analysis

To further confirm the different strategies of the clusters, we analyzed striking behavior at two complementary levels (the strength of kinematic coupling within individual trials and the magnitude of parameter scaling across varying task demands) using a Hierarchical Multiple Factor Analysis (HMFA). This analysis used Cluster as an active categorical block to provide a classification framework, while integrating three distinct continuous data blocks: *Strike Amplitude* (*A*), *Strike Duration* (*D*), and *Impact Speed* (*IS*). By organizing the kinematic data into these functional blocks, the HMFA allowed us to determine how the coordinated variance across spatial and temporal dimensions collectively contributes to the separation of the three clusters, in order to provide a multidimensional validation of the “Inverse Duration-Amplitude,” “Temporal Invariance,” and “Late-Impulse” strategies.

**Fig. 11:**
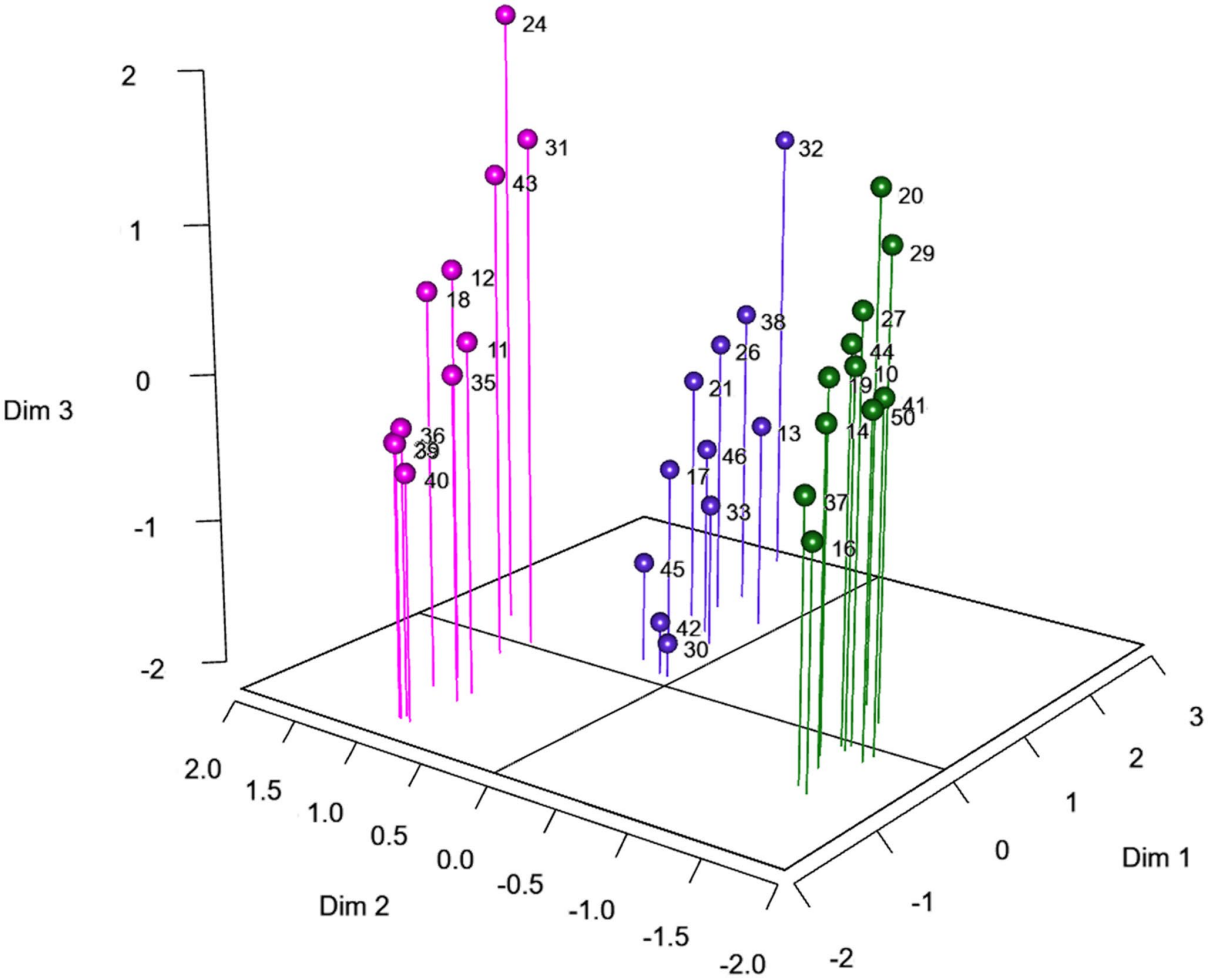
Individual coordinates in the 3D space defined by the first 3 dimensions of the HMFA (Cluster 1: green dots, Cluster 2: purple dots, Cluster 3: magenta dots)

The HMFA showed that 4 dimensions captured 88.2% of the variance, with most of that variance (77.8%) in the first 3 dimensions, as illustrated in Fig. 11. Results suggest that the first two dimensions primarily capture between-cluster differences, while Dim3 reveals structured within-cluster variability that is consistent across clusters (rather than residual noise). The clusters are regular and compact in the horizontal plane (Dim1-Dim2). Within each cluster, individuals are distributed coherently along Dim3, which captures a shared internal structure related to the combined magnitude of both *A* and *D*.

**Fig. 12:**
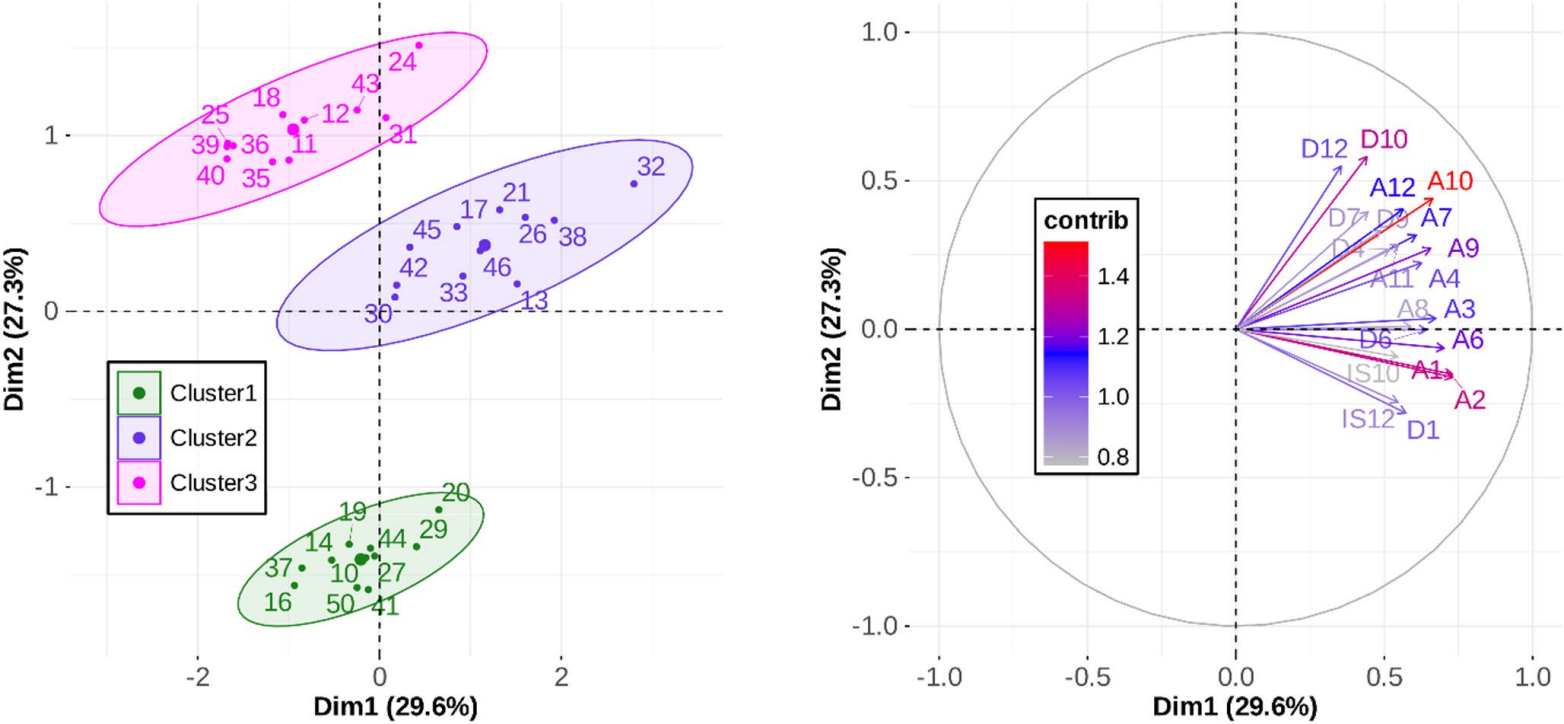
Individual coordinates (left panel) and 20 most contributory quantitative variables (%) (right panel) for the first two dimensions of the HMFA

To properly qualify each dimension, we analyzed the between- and within-cluster coordinates for each pairwise dimensional solution, along with the quantitative variables that contributed most significantly to each axis. As illustrated in the left panel of Fig. 12, the separation between clusters along Dimension 2 (Dim2, vertical axis) primarily reflects the mean correlation between *D* and *IS*. This axis effectively differentiates the clusters based on their temporal scaling rules, with Cluster 1 exhibiting a negative correlation (*r*(*D*, *IS*) = -0.44), Cluster 2 demonstrating temporal invariance (*r*(*D*, *IS*) = 0.01), and Cluster 3 showing a positive correlation (*r*(*D*, *IS*) = + 0.32). In contrast, the within-cluster dispersion along Dim2 captures inter-individual variability in baseline *D* mean. Within Cluster 1, for instance, participants 16, 41, and 50 displayed the shortest *D* mean (approximately 100 ms), whereas participant 20 exhibited the greatest within-cluster *D* mean (*M* = 172 ms). A similar spread was observed in Cluster 3, where participant 24 showed the greatest *D* mean (*M* = 231 ms) while participants 11, 35, and 40 maintained the shortest durations within the group (approximately 100 ms). The construction of Dim2 was dominated by the contributions of Cluster 1 (60.9%) and Cluster 3 (33.5%), while Cluster 2 accounted for only 5.5%. This distribution confirms that Dim2 specifically captures the divergent modulation of *D* in relation to *IS*. Finally, the categorical “Cluster” variable accounted for 88.7% of the variance in Dim2 construction, whereas the quantitative variables (*A*, *D*, and *IS*) contributed to the remaining 11.3%.

In contrast, Dimension 1 (Dim1, horizontal axis) was constructed with nearly equal contributions from both categorical and quantitative variables (53.8% and 46.2%, respectively). The right panel of Fig. 12 illustrates the contribution (in %) of each quantitative variable to the HMFA dimensions across the 12 levels of task demand. Visual inspection shows that *A* was the dominant quantitative variable for Dimension 1, accounting for approximately half of the variance explained by the quantitative block. Along this dimension, inter-individual variability reflects a compromise between within-cluster and between-cluster dispersion, as shown in the left panel of Fig. 12. Cluster 2 exhibited the greatest *A* mean (*M* = 143 mm) relative to the other clusters, which averaged approximately 125 mm. Additionally, Cluster 1 displayed the smallest within-cluster variability (*SD* = 33 mm) compared to the other clusters (*SD* approximately 47 mm). This within-cluster dispersion is clearly evident in the data from Cluster 2, where participant 32 had the largest *A* mean (*M* = 231 mm), while participants 30, 42, and 45 had the smallest mean amplitudes, averaging approximately 83 mm.

**Fig. 13:**
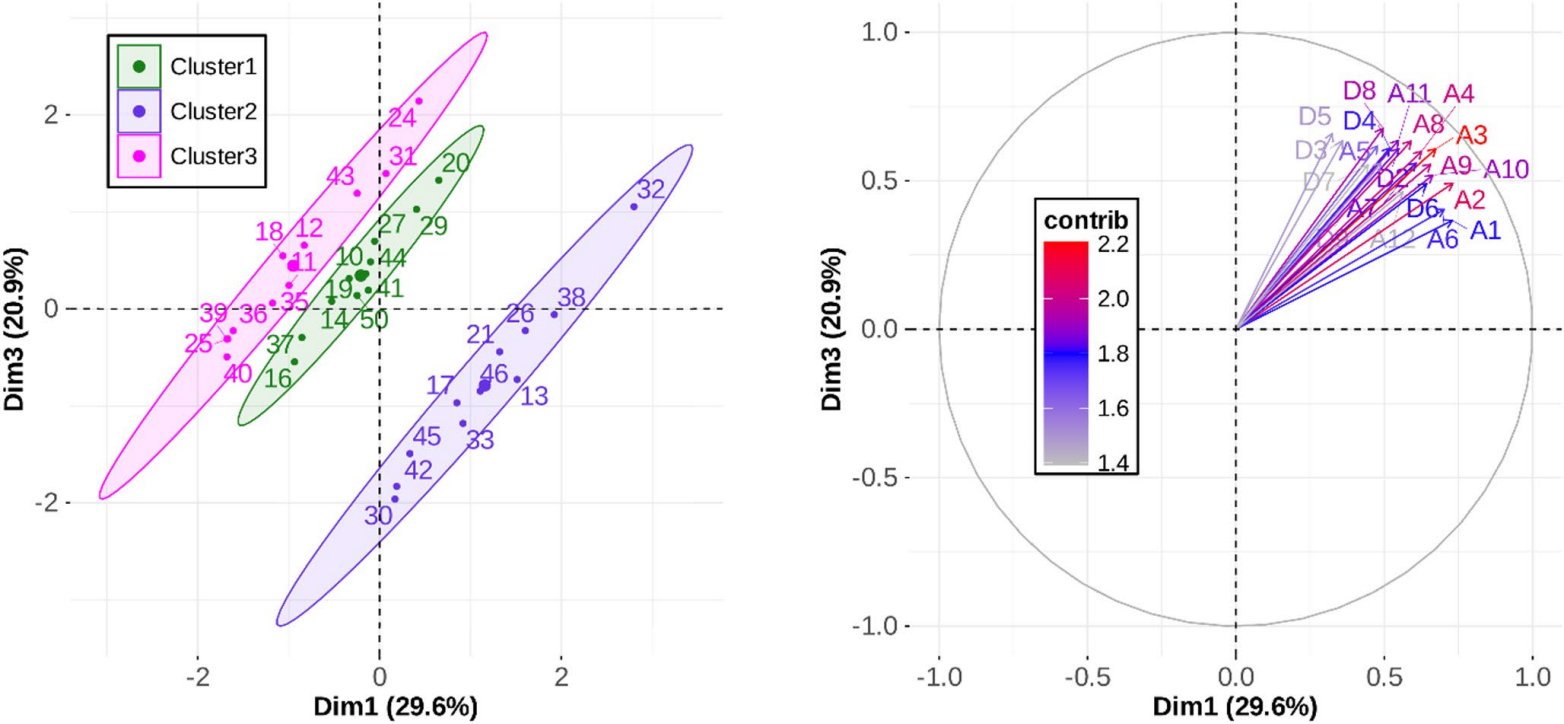
Individual coordinates (left panel) and 20 most contributory quantitative variables (%) (right panel) for Dim1 and Dim3 of the HMFA

Dimension 3 (Dim3, vertical axis in Fig. 13, left panel, and Fig. 11) was also constructed with nearly equal contributions from both categorical and quantitative variables, at 44.19% and 55.9%, respectively. Within the quantitative block, *A* and *D* were the most contributory variables, accounting for 89% of this block’s contribution to the dimension. As illustrated in the right panel of Fig. 13, Dim3 reflects the variability associated with the combined magnitude of *A* and *D*. This relationship is further evidenced by the within-cluster variability shown in the left panel of Fig. 13. For example, within Cluster 3, participant 24 exhibited the highest mean values for both parameters (*A* = 204 mm and *D* = 231 ms), whereas participant 40 showed the lowest mean values (*A* = 78 mm and *D* = 91 ms).

**Fig. 14:**
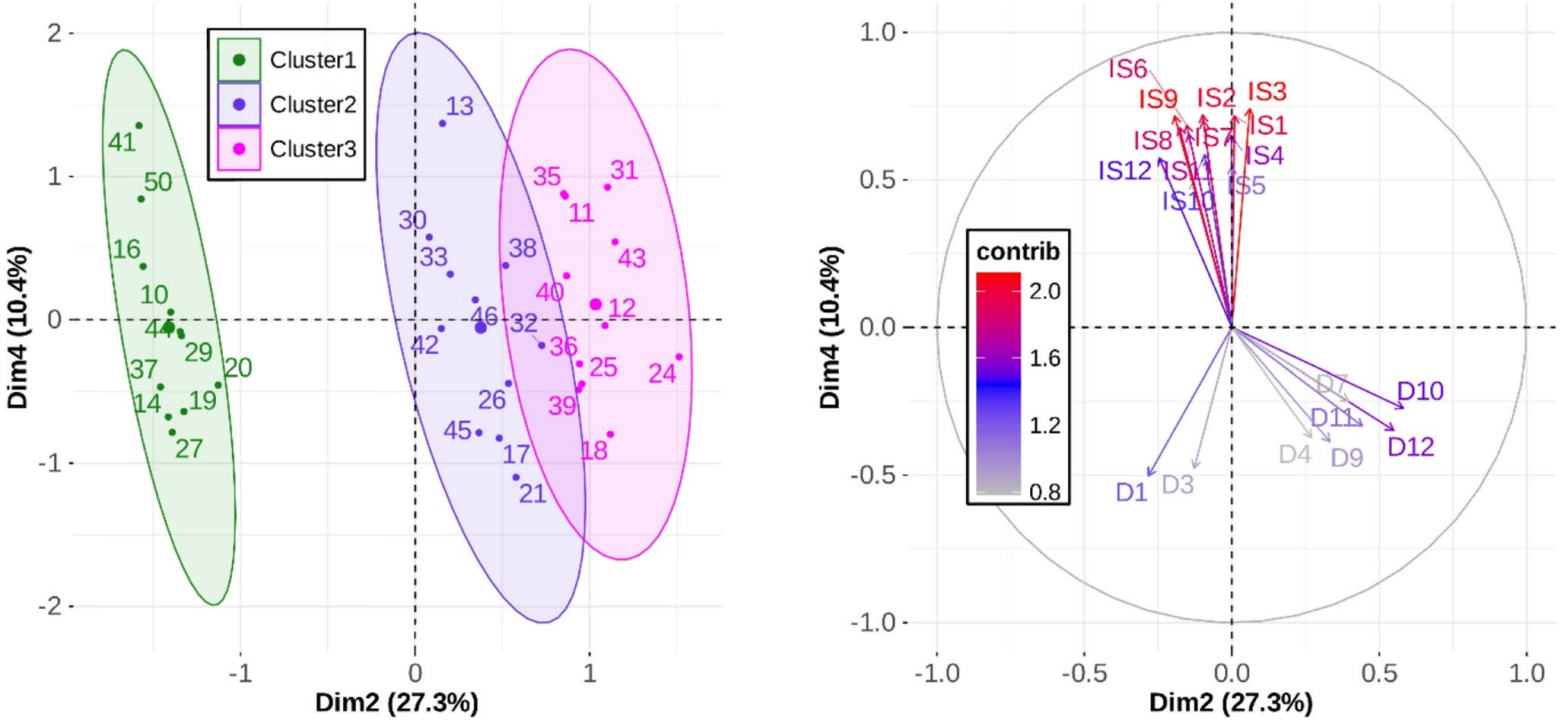
Individual coordinates (left panel) and 20 most contributory quantitative variables (%) (right panel) for Dim2 and Dim4 of the HMFA

Finally, Dimension 4 (Dim4; vertical axis in Fig. 14, left panel) was constructed almost exclusively from the quantitative blocks (96.7%). Within this contribution, *IS* accounted for approximately three-quarters of the variance, while *D* accounted for the remaining quarter, as illustrated by the contribution of these quantitative variables to Dimension 4 in the right panel of Fig. 14. The within-cluster variability along this dimension reflects differences in *IS* magnitude across participants. For instance, as shown in the left panel of Fig. 14, participant 13 within Cluster 2 exhibited the highest mean *IS* (3.24 m/s), whereas participants 17, 21, and 45 showed the lowest mean values (approximately 2 m/s).

### Complementary information for spatio-temporal strategy performance

As previously mentioned, in another article (Famié et al. 2022), we identified two primary motor coordination strategies among participants: one group primarily adjusted forearm amplitude to increase *Impact Speed*, while the other group predominantly varied wrist rotation amplitude. However, the study did not explore the spatio-temporal control of the striking movement. After examining the frequency of a particular spatio-temporal strategy (Giszter 2015) within each motor strategy cluster, we found that these strategies are independent. Indeed, if we look at the distribution of motor strategies within each spatio-temporal strategy cluster, we observe a proportionally similar distribution of individuals across the two motor strategies.

As previously mentioned, Famié et al. (2022) identified two main motor coordinations (corresponding to two different groups of participants) to perform the striking task. A re-analysis of the data shows homogeneous frequency of a given spatio-temporal strategy within each motor coordination group, suggesting that these different strategies (in terms of motor coordination or spatio-temporal regulation) are independent (see Table 2). A chi-square test of independence was performed to examine the relation between Spatio-temporal strategy and Motor Coordination strategy for the striking task. The relation between these variables was nonsignificant, χ² (2, *N* = 33) = 0.248, *p* = 0.88, Cramér’s *V* = 0.087 (negligible effect size). These results suggest that spatio-temporal strategies and motor coordination are independent, refuting our hypothesis that motor coordination depends on whether a participant prioritizes amplitude or duration.

**Table 2:** Number of participants in each motor coordination strategy according to spatio-temporal strategy clusters

	Cluster 1: Inverse Duration-Amplitude Strategy	Cluster 2: Temporal Invariance Strategy	Cluster 3:Late-Impulse Strategy	Total participants
Wrist coordination group	4	5	5	14
Forearm coordination group	7	6	6	19

## Discussion

To cope with the inherent variability in motor control, due to factors such as redundancy (Sternad 2018) and neural noise (Faisal et al. 2008), humans must process sensory information to guide movement execution, resulting in individual perceptual-motor styles (Vidal and Lacquaniti 2021). Individuals may adopt similar adaptations to environmental constraints, leading to the emergence of common perceptual-motor strategies across subgroups. For example, Maselli et al. (2017, 2019) identified consistent spatio-temporal control strategies across four distinct groups, categorized by stepping patterns during ball throwing (no-step, right-step, left-step, and double-step). Furthermore, Słowiński et al. (2016) found that when individuals attempt to reproduce another’s movement, they often replicate that person’s motor signature, sharing motor styles. In the present study, we extended this approach by analyzing the spatio-temporal signature of hand movements during a striking task in which an object slides across a surface toward a target distance. Task demand varied based on the combination of three factors (target distance, surface material, and surface slope), corresponding to a specific required optimal speed at impact to slide the object to a given position. Despite adopting one of three distinct motor strategies, participants achieved similar mean spatial accuracy in target distance, revealing different yet equally effective motor styles underlying task performance.

The motor control strategies evidenced in our behavioral data can be interpreted within the contemporary neural manifold perspective (Gallego et al. 2018; Rodriguez et al. 2024). Gallego and colleagues proposed that neural activity in primary motor cortex (M1) evolves within a stable, low-dimensional neural manifold composed of preserved “neural modes” that reflect latent population dynamics consistent across diverse motor tasks. Within this framework, the central nervous system (CNS) is thought to generate a wide range of actions not by reconfiguring the entire neural network for each task, but by flexibly combining and modulating these underlying dynamical components. Building upon this, Rodriguez et al. (2024) examined how motor behavior may be parametrically modulated within such a neural manifold using a self-paced sequential reaching task in non-human primates. In their study, target locations varied pseudorandomly in distance (5–15 cm) and angle (0–60°) relative to the previous target, inducing variability in movement direction, amplitude, and speed. Although this task design introduced multiple sources of kinematic variability, the authors noted that peak arm speed strongly correlated with target distance. To capture the underlying neural population activity, monkeys were implanted with electrode arrays in the dorsal premotor cortex (PMd) and the primary motor cortex (M1). By applying PCA to the recorded activity, the authors dissociated spatial features, represented by the geometric path of the neural trajectory in state space, from temporal features, represented by the rate at which that path is traversed. Their results suggested that, for a given movement direction, neural activity tends to follow a preserved geometric path in state space, and that variations in movement speed are primarily accounted for by the velocity of the signal along that path.

Within this conceptual framework, the three motor control strategies identified in our study may reflect different ways in which spatial and temporal aspects of motor commands are coordinated. The *Inverse Duration-Amplitude Strategy* (Cluster 1) would reflect a functional state where the spatial and temporal components are tightly coupled. Contrary to our hypothesis of a “constant amplitude” strategy, participants in this cluster increased *Impact Speed* through a reciprocal scaling: simultaneously increasing *Strike Amplitude* while decreasing *Strike Duration*. This could be related to the biomechanical constraints of the striking gesture or to a functional advantage (as reflected in the relative invariance of spatial error with increasing task demand), which characterizes the strategy adopted by Cluster 1. From a neural manifold perspective, this suggests that the CNS increases the spatial extent of the neural trajectory while concurrently increasing the traversal rate (neural speed) along that path. Such behavior is consistent with a classical scaling relationship and may reflect limited functional independence between spatial and temporal components of the underlying motor dynamics. In contrast, the *Temporal Invariance Strategy* (Cluster 2) is characterized by temporal invariance, with *Impact Speed* modulated primarily through changes in amplitude. This pattern may reflect selective scaling of the geometric extent of the neural trajectory while preserving traversal rate, consistent with the dissociation between spatial and temporal features of neural activity described by Rodriguez et al. (2024). Finally, the *Late-Impulse Strategy* (Cluster 3) suggests a more complex relationship between *Strike Amplitude*, *Strike Duration*, and *Impact Speed*, with participants increasing both amplitude and duration to achieve higher speeds. One possible interpretation is that extending both the spatial path and the temporal window permits a non-uniform traversal of the underlying neural trajectory, characterized by a late-phase acceleration. However, this interpretation remains speculative and is offered as a conceptual mapping rather than a direct inference about neural dynamics.

Although our study did not include electromyographic (EMG) recordings, these behavioral strategies can also be discussed within the framework of time-varying muscle synergies (D’avella and Lacquaniti 2013). In this view, movements are constructed from spatial synergies that specify patterns of muscle co-activation and temporal synergies that govern their timing and duration (Delis et al. 2018). This modular architecture provides the physiological basis for the independent control of movement amplitude and timing, allowing the CNS to manage the trade-off between *Impact Speed* (*IS*) and *Strike Duration* (*D*) in distinct ways. From this perspective, the *Inverse Duration-Amplitude Strategy* may reflect a rigid coupling between spatial and temporal control modules. To increase *IS*, the CNS may simultaneously scale the spatial component of the movement (increasing amplitude) and compress its temporal component (decreasing duration). This suggests a control scheme in which the two modules are governed by a single, shared scaling parameter, leading to faster strikes that are inherently shorter. In contrast, the *Temporal Invariance Strategy* may reflect greater functional independence between spatial and temporal control modules. By maintaining temporal invariance, these participants effectively stabilize the temporal component of the movement while selectively scaling its spatial component. This allows them to achieve higher *IS* through increased muscular recruitment and spatial extent without altering the movement’s temporal structure. This strategy directly exploits the dissociation described by Delis et al. (2018), treating the timing and magnitude of muscle activation as independent control variables. Finally, the *Late-Impulse Strategy* may reflect a more complex regulation of the *IS*-*D* relationship. At the muscular level, this pattern could involve an extended temporal window of activation, allowing a late-phase increase in acceleration. From a neural perspective, such behavior may arise from a non-linear modulation of motor commands, with motor unit recruitment weighted toward the final phase of the movement. The combined increase in movement duration and amplitude may thus enable a pronounced terminal impulse, thereby maximizing *IS*. A brief terminal muscular co-contraction could, in turn, increase joint impedance at contact, facilitating effective energy transfer while preserving limb stability. However, because the present analysis is based solely on kinematic measures, future studies incorporating EMG recordings will be necessary to directly assess whether such late-phase activation and changes in joint stiffness are present.

Participants in the “*Inverse Duration-Amplitude Strategy*” group employed a spatiotemporal strategy consistent with findings from repetitive movement tasks, such as the baseball swing. In these tasks, individuals adjust the timing of their movements to precisely control speed at impact (Katsumata 2007). In contrast, participants in the “*Temporal Invariance Strategy*” group regulated *Impact Speed* by varying *Strike Amplitude*. This strategy aligns with previous studies showing that adjusting movement amplitude based on target distance improves spatial accuracy (Delay et al. 1997; Hume et al. 2005). Finally, participants in the “*Late-Impulse Strategy*” group opted for expanding both spatial and temporal parameters, which serve as a functional window to facilitate a terminal acceleration burst. Rather than a uniform increase in speed, participants utilizing this strategy employ a longer, more controlled approach phase to generate a sharp, late-stage impulse. Prior studies on golf putting (Dias et al. 2014; Hasegawa et al. 2017) have shown that, in complex tasks requiring fine motor control, simultaneous modulation of multiple spatio-temporal parameters enables more precise tuning of pre-impact acceleration and velocity.

Inter-individual variability in motor strategies that yield equivalent mean performance can be understood through the concept of motor redundancy (Bernstein 1967). This redundancy enables individuals to adapt their behavior to task constraints by exploiting the available degrees of freedom to optimize movement over time (Sternad 2018). In a previous study, we identified distinct upper limb coordination strategies for controlling a striking gesture to slide an object to a target distance (Famié et al. 2022), such as increasing forearm rotation amplitude versus primarily varying wrist rotation amplitude, each reflecting different joint contributions and illustrating the principle of intrinsic redundancy in motor control (Sternad 2018). In the present study, we observed variability in the spatio-temporal characteristics of fingertip trajectories prior to impact, highlighting the role of extrinsic redundancy, that is, the multiple ways a given movement outcome can be achieved through different temporal and spatial strike parameters (Sternad 2018). Interestingly, the three identified spatio-temporal strategies were found across participants regardless of their preferred motor coordination strategy. This suggests that intrinsic and extrinsic redundancy mechanisms can be flexibly combined to achieve comparable mean performance outcomes. However, although performance for the *Inverse Duration-Amplitude Strategy* did not vary with task demand, the other two strategies tended to undershoot the target distance as task demand increased. Actually, we found that the distribution of spatio-temporal strategies was balanced within each motor coordination group. While one might expect participants favoring a wrist-dominant coordination (characterized by smaller angular amplitudes) to prefer either the *Inverse Duration-Amplitude Strategy*, which is characterized by a modulation of *Strike Duration*, whereas those using a forearm-dominant coordination (allowing for larger angular amplitude variations) would rely on a more amplitude-based strategy like the *Temporal Invariance Strategy*, this was not systematically observed. Furthermore, we expected that participants who jointly vary both *Strike Amplitude* and *Strike Duration* to regulate gesture acceleration, such as in the *Late-Impulse Strategy*, would be distributed across both motor coordination groups; this was indeed supported by our data. This dissociation indicates that coordination strategies relying on joint-level control (intrinsic redundancy) and spatio-temporal regulation of movement trajectories (extrinsic redundancy) can operate independently, further underscoring the flexibility and adaptability of the human motor system in responding to complex task demands.

A promising extension of the current work would have been to analyze inter-trial variability using an Uncontrolled Manifold (UCM) framework (Latash et al. 2007). Such an analysis would have been particularly insightful to determine which of our performance variables of interest (*Strike Duration*, *Strike Amplitude*, or *Impact Speed*) are prioritized and stabilized by motor synergies. Notably, previous research employing UCM analysis in tasks where *Impact Speed* is a critical determinant of success, such as throwing (Bennett et al. 2024) and stone knapping (Rein et al. 2013), has successfully demonstrated how the central nervous system organizes elemental variance to stabilize key performance outcomes. However, we were unable to perform a robust UCM analysis due to specific methodological constraints. The UCM approach requires a high number of repetitions per condition to ensure a reliable estimation of the covariance matrix. While our protocol utilized blocks of 10 repetitions to capture short-term behavioral adaptation to surface and distance changes (Famié et al. 2022), this trial count remains below the threshold required for stable UCM metrics (Pawłowski et al. 2021). Consequently, while the present study establishes that spatio-temporal strategies remain independent of joint-level coordination modes, future investigations with more extensive trial sets are necessary to fully characterize the synergistic structure of this object-sliding task.

To conclude, the presence of inter-individual variability in spatio-temporal motor control regardless of task demand highlights the importance of redundancy and flexibility in the human motor system. Strategic diversity among individuals to adapt to variable task demands opens new perspectives for research and practical applications in motor control. In particular, the findings make the case for customizing interventions to individual motor preferences, for example, by influencing adaptive control systems in contexts like human-robot cooperation (Peternel et al. 2016) and robot-guided practice, such as in golf putting (Bested et al. 2019).

## Data Availability

The data that support the findings of this study are available in the Zenodo repository (https://zenodo.org/records/19924581).
